# A Longitudinal Study on the Relationship of Oral Health at 4 Years of Age with That in Adulthood

**DOI:** 10.3390/dj9020017

**Published:** 2021-02-01

**Authors:** Sho Yamada, Reiko Sakashita, Mikinori Ogura, Eiko Nakanishi, Takuichi Sato

**Affiliations:** 1 Takenoyama Dental Clinic, Nissin 470-0136, Japan; info@takenoyama-dc.jp; 2 College of Nursing Art and Science, University of Hyogo, Akashi 673-8588, Japan; eiko_nakanishi@cnas.u-hyogo.ac.jp; 3 Maxillofacial Unit, Oita Oka Hospital, Oita 870-0192, Japan; mogura-clp@umin.ac.jp; 4 Division of Clinical Chemistry, Niigata University Graduate School of Health Sciences, Niigata 951-8518, Japan; tak@clg.niigata-u.ac.jp

**Keywords:** oral health, dental caries, periodontal disease, occlusion, bite-force, infant, longitudinal study

## Abstract

This longitudinal study aimed to clarify the relationship of oral health in infancy with that in adulthood among participants who were the subjects of the oral health promotion project (OHPP) conducted in Miyako Island, Okinawa Prefecture, Japan, since 1984. Twenty-seven subjects, around 35 years of age, were examined for dental caries, periodontal diseases (community periodontal index), dental plaque, occlusion, and bite-force and compared with those at 4 and 13–15 years of age. The dental caries status and maximum bite force in adulthood was significantly reflected for those at 4 and 13–15 years of age (*p* < 0.05). CPI in adulthood was related to the dental caries status at 4 and 13–15 years of age but not to the gingival score at 4 years of age, and it was weakly related to the gingival score at 13–15 years (r = 0.264, *p* > 0.05). Most of the normal occlusion at 4 years of age became normal permanent occlusion in adulthood (88.9%). Most of the cases involving the discrepancy factor retained the same condition in both the deciduous and permanent dentitions (83.3%) (*p* < 0.001). Those who participated in the OHPP soon after birth showed significantly fewer DMFT (*p* < 0.05) compared with those who did not. This study revealed that oral health at 4 years of age was related to that in adulthood, suggesting that fostering good oral health soon after birth is of great importance.

## 1. Introduction

It has been suggested that the underdevelopment of the masticatory system will increase among young Japanese population [1,2]. For example, 63.1% of young Japanese people (n = 495) have crowding of teeth [3], and Japanese children currently show a narrowing of the dental arch width as compared with those in 1964 [4], and 34.6% of children aged 3 to 5 years (n = 107) have problem in eating such as slow eating and less function [5]. The present diet, with its soft and nutritious food, bottle feeding, and long-lasting pureed baby food, has been suspected to be the cause of these problems [6]. Experiments have shown that breast-fed mice have a better developed masticatory system than bottle-fed ones. A diet of liquidized or pureed food reduces the height of the mandible, the length of the body of the mandible, and the condylar width, and widens the gonial angle [7]. It also reduces the size of the masseter and temporal muscles as well as of the salivary glands [8]. Experiments with rats have also shown that the feeding of a liquid diet after being weaned may alter the motor output of jaw and tongue muscles and obstruct the functional transition from suckling to mastication, and jaw elevators that develop without motor learning of mastication are inefficiency when performing functionally [9].

Hence, if diet can be the main cause of not only dental diseases but also insufficient development of the masticatory system, healthy oral conditions can possibly be brought about by a change in the dietary regime from a young age. Hoping to reduce the insufficient development of the masticatory system and to prevent dental problems through health education focusing on diet, we set up the Oral Health Promotion Project (OHPP) and a longitudinal study in selected districts on Miyako Island, Okinawa Prefecture, Japan, in 1984 [10]. The OHPP lasted eight years, with another nine years of follow-up, and completed in the year 2000. Positive effects of this project were reported on dental occlusion, masticatory function, and dental caries in the participants at 4 and 13–15 years of age [11,12].

After graduating from junior high school, many participants left this remote island for study or work, but some of them were expected to return for the reunion to celebrate the 20th anniversary of graduating from junior high school in Karimata on Miyako Island, when they were about 35 years old. On this and on other occasions, such as New Year’s and Homecoming, this study aimed to clarify the relationship between the oral conditions at 4 years of age (completed deciduous dentition [Hellman’s dental age, IIA]), 13–15 years old (permanent dentition [IVA]), and in adulthood.

## 2. Methods

### 2.1. Study Design

This prospective, longitudinal study was based on three examinations conducted in Ikema and Karimata district, remote Island Miyako, Okinawa, Japan.

### 2.2. The Oral Health Promotion Project (OHPP)

The Project began in April 1984. During the first eight years, the OHPP intervention focused mainly on health education, with special emphasis on diet and thrice-yearly dental examinations and treatments such as cavity restoration [10]. Participants were followed up, including an assessment of their oral health, once a year until the year 2000. Children born in the district between 1979 and 1992 were enrolled in the OHPP.

Based on previous studies [6,10,13], the guidelines for oral health promotion were developed [1]. At the first stage of the lactation period, ‘breast feeding’ was recommended; at the second step of the transition from milk to solid food, ‘natural weaning’, i.e., the introduction of food at the proper time was advised. In the third step, after approximately three years of age, when the deciduous dentition was complete, establishment of a good dietary style was suggested.

Two small communities, Ikema and Karimata, Miyako Island, Okinawa, where no regular dentist served and public health nurses requested for help in improving the poor oral condition of the population in 1984, were selected as model venues for the OHPP.

### 2.3. Subjects

The subjects who participated in the OHPP had records at two periods, namely at 4 years of age (completed deciduous dentition [IIA]) and at 13–15 years (permanent dentition [IVA]). The main targets were 39 participants born between 1982 and 1985 in Karimata, who would possibly return for the 20th anniversary reunion around 35 years of age. In addition, invitation cards were sent to the 70 expected participants to inform them of dental surveys and to request their participation. However, 46 cards (65.7%) were returned as the addresses were unknown.

### 2.4. Examinations

#### 2.4.1. Oral Health Examinations

Dentists examined each participant to determine the oral condition, including dental caries, periodontal diseases [14], dental plaque [15], and occlusion [11], according to the criteria shown in Table 1. This was done using a mouth mirror and an explorer, with careful consideration of the lighting conditions. Dentists assessed the dental caries status as intact, decayed, missing, or filled teeth, and DMF (number of decayed, missing, or filled permanent teeth) was used as an index.

The previous data at 4 years and 13–15 years of age contained the Gingival Score (GS), in which the areas of greatest gingivitis progression were scored following Dunning’s criteria [16]. For the deciduous dentition, pseudo-normal occlusion, which is a deciduous normal occlusion with closed dental arches, was used to determine occlusion [11].

#### 2.4.2. Bite-Force Measurements

The bite-force was measured using a bite-force transducer (Occluser, Nagano Seiki Co., Tokyo, Japan). The block portion of the transducer was inserted into the subject’s mouth. The bite block was positioned parallel to the occlusal plane and was centered on the mandibular second molar. Maximum bites on both sides were measured, and the maximum value was used as the maximum bite force.

### 2.5. Data Analysis Methods

The data of this study were compared with that at 4 and 13–15 years of age. The relationship of the oral condition in adulthood with that at 4 and 13–15 years of age was examined [11,12].

1. Spearman’s correlation coefficient among quantitative variables was performed to examine the relationship among variables.
2. Chi-squared tests were performed to examine the relationship among age-related occlusion.
3. In previous studies [11,12], a major improvement of oral health has been observed in children born after the OHPP started (1984). Thus, the oral conditions were compared between those born before 1984 and in or after 1984 using t-tests for quantitative variables and the Chi-squared test for nominal scale.
4. Differences in the bite force by age and sex were tested using repeated measures ANOVA with post hoc multiple comparison by Bonferroni.

Statistical analysis software (SPSS PASW Statistics 24, Tokyo, Japan) was used for testing.

### 2.6. Ethical Approval

This study was conducted in accordance with the Declaration of Helsinki, and the protocol was approved by the Research Ethics Committee of the College of Nursing Art and Science, University of Hyogo (KYOUIN20) on 13 December 2017. Written informed consent was obtained from the participants.

## 3. Results

### 3.1. Subjects’ Characteristics

The demographic data of the subjects are shown in Table 2. A total of 27 subjects (mean age, 34.5 ± 1.5 years; 66.7% male) participated in this study. As there were no significant sex differences except for the bite-force, males and females were analyzed together, except for the analysis of bite-force.

### 3.2. Longitudinal Change in Oral Conditions 

The high values of the DMF (number of decayed, missing, or filled deciduous teeth) at 4 years (11.6 ± 5.3) dropped to 5.7 ± 3.1 at 13–15 years of age, with the replacement to permanent teeth; however, it went up to 12.2 ± 5.7 around 35 years of age (Table 3). Participants born in and after 1984 had significantly lower DMF levels compared to those born before 1984 (*p* < 0.05). The CPI scores were also lower among those born in/after 1984, although the difference was not significant (Table 3).

The percentage of normal occlusion increased from 33.3% at 4 years to 44.4% around 35 years of age. Less crowding was found in those born in/after 1984 (11.1%) compared to those born before 1984 (44.4%), although the difference was not significant. More significant functional factors were found in those born in 1984 (33.3%) compared to those born before 1984 (0.0%) (*p* < 0.05) as causative factors (Table 3).

### 3.3. Longitudinal Change in the Maximum Bite Force

Significant differences were observed among all age groups and sexes (*p* < 0.001). The maximum bite force increased significantly with age and the sex gap, and it was the largest around 35 years of age (Table 4).

### 3.4. Correlation among Variables

The dental caries status and the maximum bite force in adulthood significantly reflected those at 4 and 13–15 years of age (Table 5). The CPI in adulthood was related to the DMF and number of untreated teeth at 4 years and the DMF at 13–15 years, whereas it was not related to the GS at 4 years of age and only weakly related to the GS at 13–15 years (Table 5). The number of untreated teeth at 4 years of age was strongly related to the DMF and the CPI in adulthood (*p* < 0.001–0.01). Dental caries, periodontal status, and dental plaque scores were significantly related to each other in adulthood (Table 5).

### 3.5. Distribution of Occlusion at 4 Years of Age and in Adulthood 

Most of the subjects with normal occlusion at 4 years of age (88.9%) had normal permanent occlusion in adulthood, whereas only 16.7% of the subjects with pseudo-normal deciduous occlusion had normal permanent occlusion (Table 6).

Most of the cases involving the discrepancy factor (83.3%) retained the same conditions in both the deciduous and permanent dentitions (*p* < 0.001) (Table 7). Two deciduous open-bite malocclusions, which were caused by thumb-sucking, disappeared in the permanent dentition, since the habit stopped with the eruption of the permanent teeth. One permanent posterior cross-bite malocclusion seemed to be caused by a tongue habit (Table 7).

## 4. Discussion

Dental caries and periodontal diseases are the two most important causes for extraction in Japan [18]. Dental caries develops in both the crowns and roots of teeth, and it can arise in early childhood as aggressive tooth decay affecting the deciduous teeth of infants [19]. In the present study, as in a previous study [12], differences in dental caries prevalence were found in those born before the onset of the OHPP compared to those born after the onset of the OHPP, and these conditions continued through adolescence (age 13–15 years) into adulthood (age 35 years) (Table 3).

The causes associated with early childhood caries are multifactorial, including infant feeding habits, dietary habits, consumption of sugary foods, oral hygiene procedures, use of fluoride, and psycho-social factors [20]. Among them, Sheiham and James [21] indicated that the consumption of sugars was a major etiological factor for dental caries. In the present study, those born after 1984 had good eating habits, such as less snack intake and greater intake of fiber-rich food [12], and this may have reflected in the low caries rate in adults born after 1984.

Several recent studies have provided evidence suggesting that periodontitis may increase the risk of systemic disorders such as cardiovascular diseases and premature low birth weight infants [22]. As oral conditions affect systemic conditions, such as atherosclerotic vascular, pulmonary disease, diabetes, pregnancy, low birth weight, osteoporosis, and kidney diseases either coincidentally or causally [23], it is very important to maintain good oral health throughout life. In this study, the gingivitis status at 4 years of age and at middle school correlated strongly with dental caries from the age of 4 to adulthood and seemed to reflect the oral hygiene status, but was not related to the CPI status of adults (Table 5). Conversely, the caries status at 4 years of age showed a strong correlation with the periodontal disease status in adults. Although the disease states of gingivitis and periodontal disease in young people are suggested to be different [24], maintaining a caries-free oral condition soon after birth may reduce the risk of periodontal disease in the future.

This study revealed that oral conditions in childhood were strongly related with those in adulthood (Table 3 and Table 5). Many studies indicate that the caries status in the deciduous dentition is related to that in the permanent dentition, although the subjects’ age was under 20 years in most of the studies [25,26,27]. There are a few longitudinal studies from childhood to adulthood, although epidemiological studies suggest that gingivitis is common in children, and the untreated cases may progress to severe breakdown of the periodontium and loss of teeth in adults [28]. Prevention should start when deciduous teeth change into permanent teeth. However, this study confirms that the condition of the deciduous teeth is passed on to the permanent teeth (Table 5). This is because the oral condition is greatly influenced by diet. Previous studies comparing the secular changes in the rates of carious teeth and the prevalence of malocclusion of discrepancy type showed that both would be expected to grow in severity as the economy progressed, with a resultant influence on dietary regimes [3,10,29,30]. Fiber-rich food has been seen to be most effective in dealing with this problem, as it produces a greater load on the jaws during mastication and promotes a self-cleansing system, reducing oral pollution [30,31]. The two main points of the OHPP dietary instructions after transition to solid food were: 1) stop drinking large amounts of high-calorie drinks like juice, especially before meals; and 2) eat fiber-rich food such as vegetables at all meals. The consumption of high-calorie drinks reduce appetite and result in not eating enough fibrous vegetables. [12]. As a result of inadequate meals, infants soon become hungry again and more juice and snacks are given. To break this vicious circle, mothers must regulate their infants’ and children’s intake so that they have empty stomachs at mealtimes. This promotes the development of the chewing system, chewing ability, and salivary secretion. Both brushing of teeth and the application of fluoride reduce dental caries [32,33]. However, it is not expected to have significant effects on the development of oral morphology and function. Therefore, we focused on dietary instructions and did not introduce fluoride mouth rinses.

Previous studies showed a decrease in crowding with the type of discrepancy, and dental caries could be seen among those born after the establishment of the OHPP [11,12]. There was less improvement in children born in 1979–1980, whose deciduous dentitions were already fully developed at the beginning of the project. Considering that the DMF improved significantly, according to the findings of this study (Table 3), the developmental “direction” and risk of dental diseases would be difficult to change with increasing age. Children’s dietary intake has been shown to be strongly associated with their mother’s dietary intake [34], and the children’s own dietary habits develop early in life [35,36]. Thus, the suckling and weaning stages seem to be the key periods.

Experiments on mice have shown that the masticatory muscles and jaw bone in breast-fed mice developed better than those in mice fed maternal milk through artificial teats [37]. In human babies, the activity of the masticatory muscles was reduced in bottle-fed babies compared to that in breast-fed babies [6,38]. Rats or mice fed on soft or liquid food showed a lower percentage of oxidative fibers in the ascending rami, and it has been demonstrated that altered muscular function can influence craniofacial morphology [8,39,40]. A constant liquid diet reduces the size of the masseter and temporal muscles, and even of the salivary glands. It also affects the chewing pattern [8,9]. Several experiments on rats have shown that insufficient mechanical stress in the growth process results in the underdevelopment of the masticatory system, the weight of the masseter muscles, and the size of the jaw bone and condylar head [41,42,43].

Based on the above findings, we recommend breast feeding and natural weaning processes, such as baby-led weaning [44], through the OHPP. The main dietary instructions in the OHPP, as described above, are important not only for the masticatory system but also for the life-long health of the whole body. Experiences in early life influence later dietary regimes [45] and could help decrease metabolic risk factors leading to obesity and obesity-related diseases [46,47,48]. In this study, we tried to confirm the relationship between feeding and weaning processes, but in vain, due to the lack of records.

One of the limitations of the present study was the small sample size, which was a consequence of the relatively small number of participants in the OHPP, as most of the young people moved out of the region in search of education and jobs, and it was difficult to track them. There was also sex bias, as female candidates tended to be less likely to participate in this research because they were too shy to let their mouth be examined. Although there have been significant advances in treatment methods over the past 30 years, the diagnostic criteria for health screening have not changed. Thus the same criteria were used in this study.

## 5. Conclusions

The dental caries status and maximum bite force in adulthood was significantly reflected for those at 4 and 13–15 years of age. Periodontal diseases in adulthood was related to the dental caries status at 4 and 13–15 years of age but not to the gingival score at 4 years of age, and it was weakly related to the gingival score at 13–15 years. Most of the normal occlusion at 4 years of age became normal permanent occlusion in adulthood. Most of the cases involving the discrepancy factor retained the same condition in both the deciduous and permanent dentitions. Those who participated in the OHPP soon after birth showed significantly fewer DMF compared with those who did not. Thus, this study revealed that oral health at 4 years of age was related to that in adulthood, suggesting that fostering good oral health and dietary habits soon after birth is of substantial importance.

## Figures and Tables

**Table 1:** Assessment scores for oral conditions.

Community Periodontal Index (CPI) [14]
A special probe was used to determine the probing depth, bleeding response, and presence of calculus. Six areas were measured (upper and lower central incisors, and the upper and lower, left and right first molars) and the highest applicable number was used.
0: Healthy periodontal tissues
1: Bleeding after gentle probing
2: Supragingival or subgingival calculus or defective margin of filling or crown
3: 4 mm or 5 mm periodontal pocket
4: 6 mm or deeper pathologic periodontal pocket
Dental plaque index (DPI)
The teeth with the most plaque were scored according to Greene & Vermillion’s criteria [15].
0: No plaque present
1: Plaque covering not more than one-third of the exposed tooth surface
2: Plaque covering more than one third, but not more than-two thirds of the exposed tooth surface
3: Plaque covering more than two-thirds of the exposed tooth surface
Categories of occlusion [17]
Each occlusion was examined and classified into one of the following categories:
(1) Normal occlusion: The upper and lower molars were in a relationship whereby the mesiobuccal cusp of the upper molar occluded in the buccal grove of the lower molar and the teeth were arranged in a smoothly curving line of occlusion.
(2) Crowding: Crowded arrangement of the teeth.
(3) Maxillary protrusion: Protruding anterior maxillary teeth.
(4) Anterior cross-bite: Reversed occlusion of the anterior teeth, i.e., one or more mandibular teeth biting in front of the maxillary teeth.
(5) Open bite: A partial gap of 2 mm or more between the anterior maxillary and mandibular teeth when in occlusion.
(6) Others: Occlusion that does not meet the above criteria
Causative factors
Four main causative factors were seen in the cases of malocclusion, and these were used in combination to describe each case (e.g., anterior cross-bite with skeletal discrepancy factors):
(1) Skeletal factor: Skeletal imbalance in size and/or shape between the maxilla and mandible.
(2) Functional factor: Functional interference in occlusion, e.g., abnormal movement of the mandible.
(3) Discrepancy factor: Tooth-to-denture base discrepancy.
(4) Habitual factor: Thumb sucking or other habit.

**Table 2:** Subjects’ characteristics.

Variable		
Age in years (Mean (SD))	34.5 (1.5)	
Sex (n, %)	Male	18 (66.7%)
	Female	9 (33.3%)
Employment (n, %)	Full-time	23 (85.1%)
	Part-time	2 (7.4%)
	Missing	2 (7.4%)

**Table 3:** Longitudinal change in oral conditions.

Variables	4 Years’ Old n = 27	13–15 Years’ Old n = 27	Adulthood n = 27	Adulthood, Born before 1984, n = 18	Adulthood, Born in/after 1984, n = 9
dmf, DMF Mean (SD)	11.6 (5.3)	5.7 (3.1)	12.2 (5.7)	14.2 (5.3) *	8.3 (4.7) *
Number of untreated teeth, Mean (SD)	5.0 (4.7)	0.9 (1.7)	1.6 (2.7)	1.6 (1.9)	1.6 (4.1)
GS < 15 years, CPI at 35 years, Mean (SD)	1.1 (0.5)	1.2 (0.6)	1.9 (1.2)	2.1 (1.2)	1.3 (1.3)
DPI Mean (SD)	0.6 (0.6)	0.9 (0.5)	0.9 (0.7)	0.9 (0.6)	0.9 (0.8)
Occlusion			(1)	(1)	
Normal	9 (33.3%)	10 (37.0%)	12 (44.4%)	8 (44.4%)	4 (44.4%)
Pseudo normal	13 (48.1%)	-	-	-	-
Crowding	0 (0.0%)	10 (37.0%)	9 (33.3%)	8 (44.4%)	1 (11.1%)
Maxillary protrusion	0 (0.0%)	2 (7.4%)	2 (7.4%)	0 (0.0%)	1 (11.1%)
Anterior cross-bite	3 (11.1%)	5 (18.5%)	1 (3.7%)	0 (0.0%)	2 (22.2%)
Others	2 (7.4%)	0 (0.0%)	1 (3.7%)	0 (0.0%)	1 (11.1%)
Causative factors					
Skeletal	1 (3.7%)	2 (7.4%)	0 (0.0%)	0 (0.0%)	0 (0.0%)
Functional	2 (7.4%)	5 (18.5%)	3 (12.0%)	0 (0.0%)	3 (33.3) *
Discrepancy	14 (51.9%)	14 (51.9%)	12 (40.7%)	8 (44.4%)	3 (33.3)
Habitual	2 (7.4%)	1 (3.7%)	1 (8.0%)	0 (0.0%)	1 (11.1%)

* A significant difference *p* < 0.05 was observed between the two groups: born before and in/after 1984. (1) Two cases were excluded in which normal occlusion was achieved after orthodontic treatment.

**Table 4:** Longitudinal change in maximum bite force.

Max Bite Force (kg)	4 Years Old		13–15 Years Old		Adulthood		Adulthood, Born before 1984		Adulthood, Born in/after 1984	
	n	Mean (SD)	n	Mean (SD)	n	Mean (SD)	n	Mean (SD)	n	Mean (SD)
Male	18	15.9 (7.3)	18	44.3 (15.9)	18	78.5 (22.5)	10	78.6 (23.8)	8	78.4 (22.3)
Female	9	9.6 (5.4)	9	38.6 (5.8)	9	38.2 (12.6)	7	35.7 (11.4)	1	55.3 (-)

Repeated measures ANOVA *p* < 0.001, sex factor *p* < 0.001.

**Table 5:** Correlation among variables.

		Adulthood				
		DMF	Untreated Carious Teeth	CPI	DPI	Maximum Bite Force
Age 4 years						
	DMF	0.504 **	0.414 *	0.392 *	0.220	−0.104
	Untreated carious teeth	0.610 **	0.256	0.675 ***	0.064	−0.133
	GS	0.579 **	0.163	0.073	0.143	−0.297
	DPI	0.068	0.167	0.078	0.020	−0.083
	Max bite force	−0.230	−0.014	−0.317	−0.031	0.443 *
Age 13–15 years						
	DMF	0.651 **	0.378	0.447 *	0.248	−0.289
	Untreated carious teeth	0.501 **	0.492 **	0.298	0.592 **	0.057
	GS	0.537 **	0.393 *	0.264	0.504 **	−0.156
	DPI	0.447 *	0.305	0.264	0.510 **	0.078
	Max bite force	−0.297	−0.172	−0.319	0.044	0.451 *
Adulthood						
	DMF		0.504 **	0.580 **	0.413 *	−0.094
	Untreated carious teeth			0.536 **	0.551 **	−0.173
	CPI				0.534 **	−0.200
	DPI					0.110

CPI: Community Periodontal Index, DPI: Dental plaque index, GS: Gingival Score. * *p* < 0.05, ** *p* < 0.01. *** *p* < 0.001.

**Table 6:** Distribution of occlusion at 4 years of age and in adulthood.

		Adulthood					
Age 4 Years		Normal	Crowding	Maxillary Protrusion	Anterior Cross-Bite	Others	Total
Normal	n	8	1	0	0	0	9
	%	88.9%	11.1%	0.0%	0.0%	0.0%	100.0%
Pseudo normal	n	2	8	1	0	1	12
	%	16.7%	66.7%	8.3%	0.0%	8.3%	100.0%
Maxillary protrusion	n	0	0	0	2	0	2
	%	0.0%	0.0%	0.0%	100.0%	0.0%	100.0%
Open bite	n	2	0	0	0	0	2
	%	100.0%	0.0%	0.0%	0.0%	0.0%	100.0%

χ2 test, *p* < 0.001.

**Table 7:** Distribution of causative factors at 4 years of age and in adulthood (around 35 years).

		Adulthood							
		Skeletal		Functional		Discrepancy ***		Habitual	
Age 4 Years		−	+	−	+	−	+	−	+
Skeletal	−	24	0						
	%	100.0%	0.0%						
	+	1	0						
	%	100.0%	0.0%						
Functional	−			22	0				
	%			100.0%	0.0%				
	+			2	1				
	%			66.7%	33.1%				
Discrepancy	−					12	1		
	%					93.3%	7.7%		
	+					1	10		
	%					16.7%	83.3%		
Habitual	−							22	1
	%							95.1%	4.3%
	+							2	0
	%							100.0%	0.0%

*** A significant relationship was observed between the discrepancy type at 4 years old and that in adulthood (*p* < 0.001). n = 26; Two were excluded because of orthodontic treatment.
